# The Cost of Gaps in Care: A Case of Lidocaine Toxicity and Postpartum Seizures

**DOI:** 10.7759/cureus.76614

**Published:** 2024-12-30

**Authors:** Jenny Lu, Nicole Pancotto, Alice Huang, Roxana Lazarescu

**Affiliations:** 1 Medicine, Touro College of Osteopathic Medicine, New York, USA; 2 Internal Medicine, Touro College of Osteopathic Medicine, New York, USA; 3 Internal Medicine, Wyckoff Heights Medical Center, New York, USA

**Keywords:** healthcare inequities, limited prenatal care, local anesthetic systemic toxicity, obstetric emergencies, postpartum complications, postpartum seizures, preeclampsia, prenatal care disparities, reproductive health disparities, socioeconomic barriers

## Abstract

This report describes the case of a 20-year-old Spanish-speaking female at 39 weeks gestation who experienced a generalized seizure immediately after lidocaine administration for a labial fold episiotomy repair following a complicated vaginal delivery. With limited prenatal care, language barriers, and socioeconomic challenges, the patient required intubation and intensive care unit (ICU) transfer for management. Extensive workup ruled out common causes, and the likely diagnosis was an atypical presentation of preeclampsia. The case highlights the impact of healthcare disparities and systemic inequities on obstetric outcomes, emphasizing the need for early prenatal care, equitable healthcare access, and advocacy for underserved populations.

## Introduction

Local anesthetics block pain primarily by preventing sodium influx into the axon. Local Anesthetic Systemic Toxicity (LAST) is a potential complication that can occur with any local anesthetic and any route of administration. There are several risk factors for LAST including age extremes, cardiac disease, renal insufficiency, hepatic disease, pregnancy, carnitine deficiency, and block site [1]. Toxicity occurs when the anesthetic agent affects the central and cardiovascular nervous systems. The signs and symptoms of LAST are highly variable. The classic presentation of LAST includes a series of progressively worsening neurologic symptoms and signs shortly after the injection of local anesthesia eventually causing seizures, coma, and in extreme cases cardiovascular collapse [2]. Intravenous lipid emulsion is the established method of treatment for acute LAST [3]. It works by sequestering lipophilic toxins from the heart and brain mainly to the liver. Additionally, it works by a possible cardiotonic effect related to changes in fatty acid processing [1,3].

Eclampsia is the occurrence of a new-onset, generalized, tonic-clonic seizure in a patient with preeclampsia or gestational hypertension. The pathophysiology of eclampsia involves systemic endothelial dysfunction, vasospasm, and altered cerebral perfusion predisposing the patient to seizures. The tonic-clonic phase of an eclamptic seizure usually resolves within 2 to 3 minutes. At this time, patients are treated with magnesium sulfate which acts to prevent recurrent seizures by stabilizing neuronal membranes and reducing excitatory neurotransmitter release [4]. Blood pressure is controlled with antihypertensives to prevent further complications such as stroke. Pulmonary embolism (PE) was also considered in this case due to its association with seizures in the context of hypoxia or circulatory compromise. Pregnancy and the postpartum period significantly increase the risk for venous thromboembolism, including PE, due to a hypercoagulable state, venous stasis, and vascular endothelial injury [5]. The management of PE typically involves anticoagulation with low-molecular-weight heparin [6]. Prompt diagnosis and treatment are essential to improve outcomes.

The overlapping features of LAST and eclampsia, such as seizures, complicate the diagnostic process. However, certain clinical features can help distinguish between the two. In LAST, seizures are often preceded by neurologic symptoms such as mental status changes [1,6]. In contrast, eclampsia is often associated with systemic features of preeclampsia, including hypertension, proteinuria, and end-organ dysfunction. 

This case underscores the diagnostic complexity of managing overlapping conditions such as LAST, eclampsia, and other postpartum complications like PE. The shared clinical features, including seizures, necessitate a thorough and systematic approach to identifying the underlying etiology. By incorporating the patient's clinical presentation, risk factors, and timing of symptoms, healthcare providers can distinguish between these conditions and implement appropriate management strategies. This case serves as a basis for exploring the interplay of these diagnoses in postpartum patients and highlights the critical need for multidisciplinary care to optimize outcomes.

## Case presentation

A 20-year-old G1P0 female with no significant past medical history presented to the emergency department (ED) with uncontrolled seizures following the delivery of a 39-week pregnancy. The pregnancy had been uncomplicated until delivery, which was notable for a narrow birth canal requiring an episiotomy and 10 mL of 2% lidocaine injected locally at the labial folds for sutures. The estimated blood loss during delivery was 400 mL. The patient had refused neuraxial anesthesia and instead underwent delivery with oxytocin infusion and lidocaine for local anesthesia. Shortly after the lidocaine injection, she experienced a seizure, prompting her transfer to the intensive care unit (ICU).

In the ICU, a Ceribell monitor confirmed status epilepticus. The patient was intubated for airway protection, complicated by aspiration during the procedure. Initial management included lorazepam 8 mg, magnesium sulfate 6 g, propofol, and levetiracetam infusion. The primary differential diagnoses were LAST due to lidocaine and atypical eclampsia. The patient presenting with seizures, neurological symptoms of altered mental status, confusion, and agitation postpartum raises suspicion for atypical eclampsia. The seizure activities after the administration of lidocaine lead to the differential of LAST. Lipid emulsion therapy was initiated for suspected LAST, and magnesium sulfate infusion was started to address the possibility of eclampsia.

**Figure 1:**
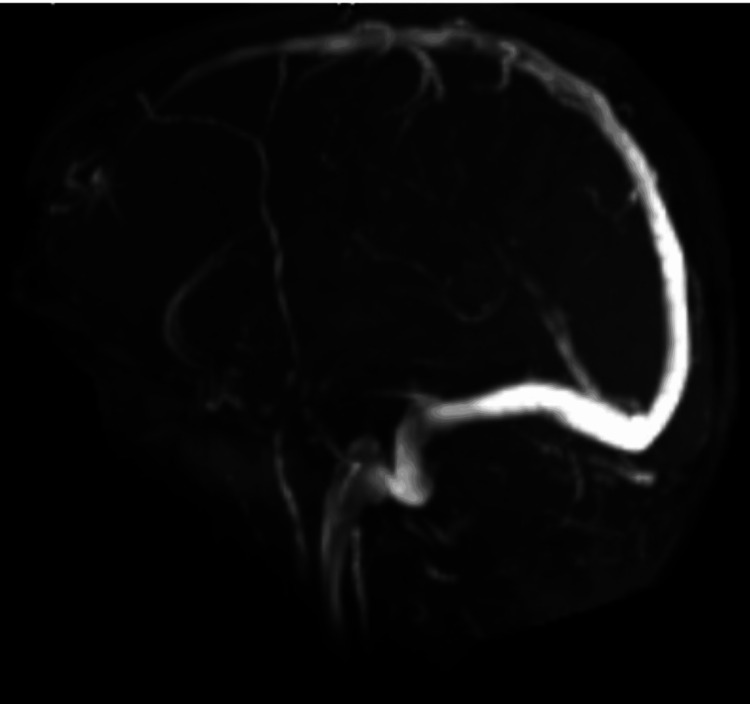
Magnetic resonance venogram shows no abnormalities in blood flow

**Figure 2:**
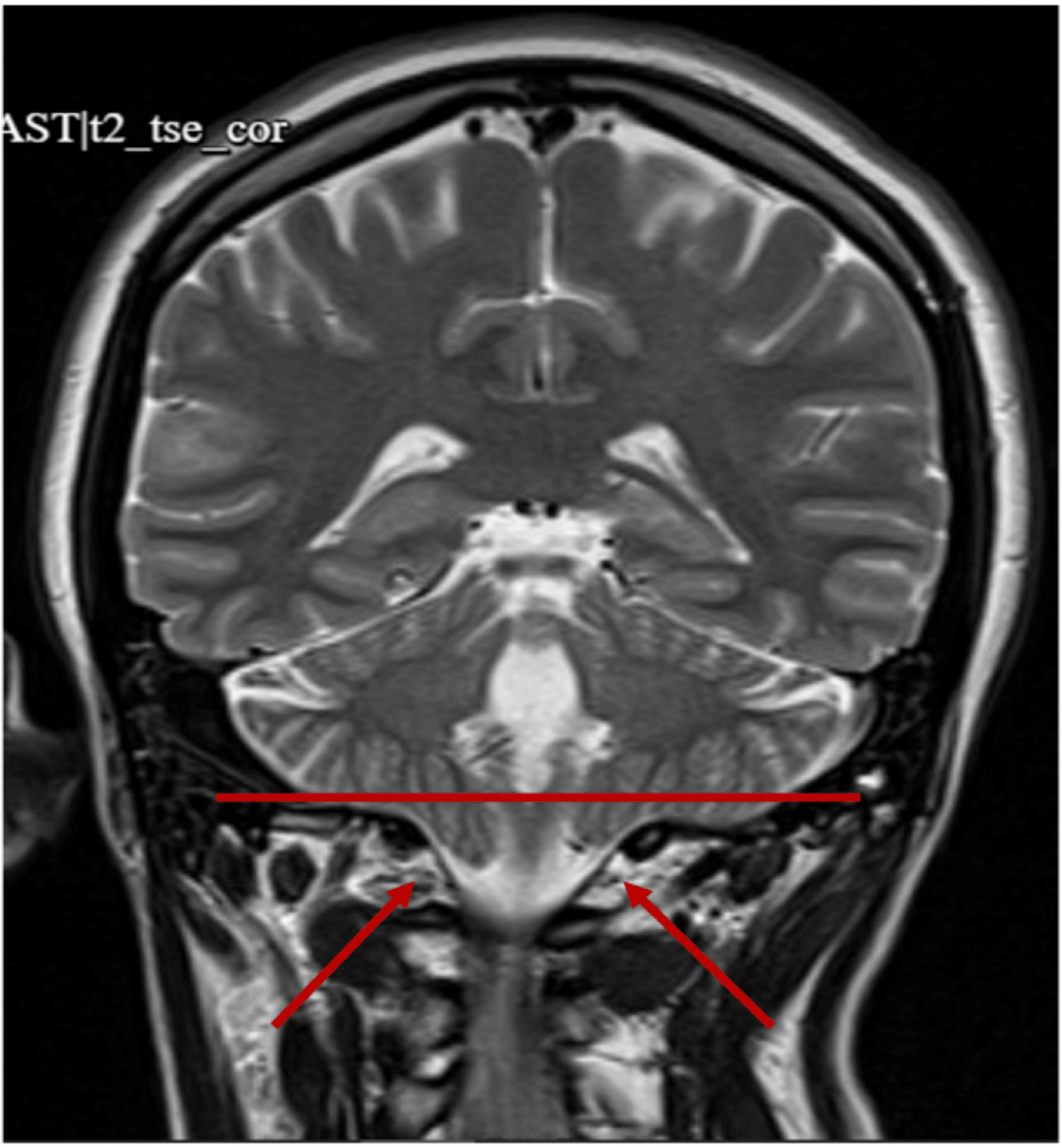
MRI of the brain The red arrows point out incidental finding of a low-lying right cerebellar tonsil extending 3 mm below the foramen magnum and mild paranasal sinus disease. MRI, magnetic resonance imaging.

Upon transfer to the ICU, the patient remained intubated and sedated on propofol. The neurology team was consulted and an order for continuous electroencephalogram (EEG) monitoring was in place and showed no additional seizures. Magnetic resonance imaging (MRI) and magnetic resonance venography (MRV) results were inconclusive for seizures, and the images did show an incidental finding of a low-lying right cerebellar tonsil extending 3 mm below the foramen magnum and mild paranasal sinus disease. Collaborative efforts from interdisciplinary teams concluded that this was not the source of her seizures. Additionally, laboratory findings revealed stable hemoglobin levels at 10.1 g/dL, and urine analysis indicated sterile pyuria with elevated white blood cells (WBCs). Empiric broad-spectrum antibiotics with piperacillin-tazobactam were initiated due to the aspiration event and suspected pulmonary consolidation seen on a chest X-ray. The ventilator settings 14/400/5/100 indicate a respiratory rate (RR) of 14 breaths per minute, a tidal volume (TV) of 400 mL, a positive end-expiratory pressure (PEEP) of 5 cm H_2_O, and a fraction of inspired oxygen (FiO_2_) of 100%, which the patient remained comfortable on with no seizure activities throughout the night and day (Figures 1, 2). 

At an RR of 14, this setting is used to ensure adequate ventilation and to prevent hypercapnia, especially important in a patient who has experienced a seizure and may have compromised respiratory drive or function. The TV of 400 mL corresponds to a lung-protective strategy, aiming for a TV of 6-8 mL/kg of predicted body weight to minimize the risk of ventilator-induced lung injury. For a typical adult female, 400 mL is within this range. PEEP of 5 cm H_2_O helps to prevent atelectasis and maintain alveolar recruitment, which is crucial in ensuring adequate oxygenation and preventing hypoxemia. FiO_2_ is set at 100% to ensure adequate oxygenation following a seizure and during the immediate post-intubation period. This is particularly important in the context of potential hypoxemia following a seizure and the need for rapid stabilization. The patient remained on magnesium sulfate infusion, and the levels showed 6.1 mg/dL which is within the therapeutic range ordered by the obstetric team for the treatment of eclampsia. 

By hospital day 3, the patient did not have any episodes of seizure activity, and she was extubated after completing 48 hours of magnesium sulfate therapy. Neurology and the intensivist team evaluated the patient and determined that her presentation was most consistent with atypical eclampsia, although LAST had been addressed with lipid emulsion therapy. She remained seizure-free on levetiracetam 750 mg every 12 hours and required no further lorazepam for breakthrough seizures. Additional history obtained from the family revealed inadequate prenatal care, with the patient only beginning obstetric visits at five months of gestation, but no reported complications during the pregnancy.

On hospital day 4, the patient was transferred to the labor and delivery unit on 2 L of oxygen via nasal cannula. She was reunited with her newborn and discharged shortly thereafter in stable condition. She was advised to follow up with the neurology clinic and to abstain from driving for one year.

## Discussion

This case underscores the challenges of managing obstetric emergencies in underinsured patients with limited prenatal care. Our patient is a Medicaid recipient, who lives in an encatchment area and her closest hospital is in an area with limited resources. We believe that the patient delayed initiating prenatal care until five months of gestation, potentially due to socioeconomic and documentation barriers, although she declined to disclose specific reasons. This delay may have prevented early detection and management of preeclampsia risk factors, such as treatment with low-dose aspirin starting at 12 weeks per USPSTF (U.S. Preventive Services Task Force) guidelines [7]. 

Preeclampsia is a significant health concern for pregnant women, affecting 2% to 8% of pregnancies globally and contributing to maternal and infant morbidity and mortality. In the United States, it is responsible for approximately 15% of preterm births [8]. This condition is characterized by the onset of hypertension (blood pressure >140/90 mm Hg) and proteinuria (≥0.3 g of protein in a 24-hour urine sample) during the second half of pregnancy (after 20 weeks). In cases where proteinuria is absent, preeclampsia is diagnosed if hypertension is accompanied by one or more of the following: thrombocytopenia, impaired liver function, renal insufficiency, pulmonary edema, or neurological symptoms such as cerebral or visual disturbances [9]. 

The patient’s postpartum seizure was likely multifactorial, with LAST as the probable trigger given that peripartum women are at an increased risk for toxicity due to pregnancy-related physiological changes. However, preeclampsia, a condition affecting 4% of pregnancies, remained a key consideration given its commonality and potential for atypical presentations. Language and cultural barriers may have further impeded her access to comprehensive prenatal care and advocacy during labor and delivery.

Healthcare disparities disproportionately affect minority and underinsured populations, often resulting in adverse maternal and neonatal outcomes. This case highlights the importance of addressing systemic barriers and maintaining vigilance in detecting and managing atypical presentations.

## Conclusions

The successful stabilization and discharge of this patient illustrate the value of comprehensive, multidisciplinary care. However, her experience serves as a stark reminder of the broader implications of healthcare inequities. In light of current political restrictions on women’s reproductive health, providers must prioritize early detection of complications and advocate for marginalized populations to ensure no patient "slips through the cracks.”
